# Caudal Epidural Block: An Updated Review of Anatomy and Techniques

**DOI:** 10.1155/2017/9217145

**Published:** 2017-02-26

**Authors:** Sheng-Chin Kao, Chia-Shiang Lin

**Affiliations:** Department of Anesthesiology, Mackay Memorial Hospital, Taipei, Taiwan

## Abstract

Caudal epidural block is a commonly used technique for surgical anesthesia in children and chronic pain management in adults. It is performed by inserting a needle through the sacral hiatus to gain entrance into the sacral epidural space. Using conventional blind technique, the failure rate of caudal epidural block in adults is high even in experienced hands. This high failure rate could be attributed to anatomic variations that make locating sacral hiatus difficult. With the advent of fluoroscopy and ultrasound in guiding needle placement, the success rate of caudal epidural block has been markedly improved. Although fluoroscopy is still considered the gold standard when performing caudal epidural injection, ultrasonography has been demonstrated to be highly effective in accurately guiding the needle entering the caudal epidural space and produce comparative treatment outcome as fluoroscopy. Except intravascular and intrathecal injection, ultrasonography could be as effective as fluoroscopy in preventing complications during caudal epidural injection. The relevant anatomy and techniques in performing the caudal epidural block will be briefly reviewed in this article.

## 1. Introduction

The caudal epidural block involves placing a needle through the sacral hiatus to deliver medications into the epidural space. This approach to the epidural space is not only widely used for surgical anesthesia and analgesia in pediatric patients but also popular in managing a wide variety of chronic pain conditions in adults.

The caudal epidural block was first introduced as a landmark-based, blind technique. In children, the successful rate with the blind technique is above 96% [1, 2]. In adults, however, it was only 68–75% even in the experienced hands [3–5]. With the advent of imaging technology, fluoroscopy and ultrasonography have been increasingly used to guide caudal epidural block. In this review, we will overview recent advancement in our understanding of relevant anatomy and development of imaging guided techniques in adults.

## 2. Anatomy

**Figure 1:**
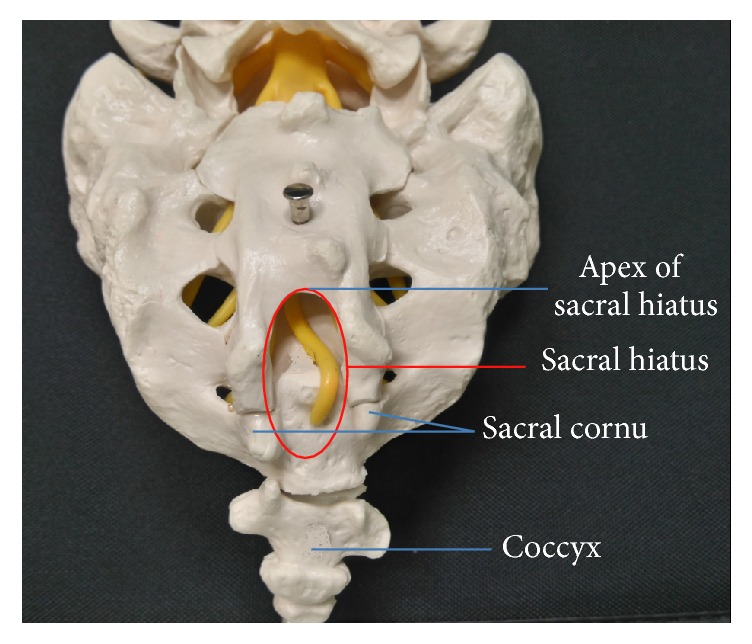
Posterior view of sacrum.

**Figure 2:**
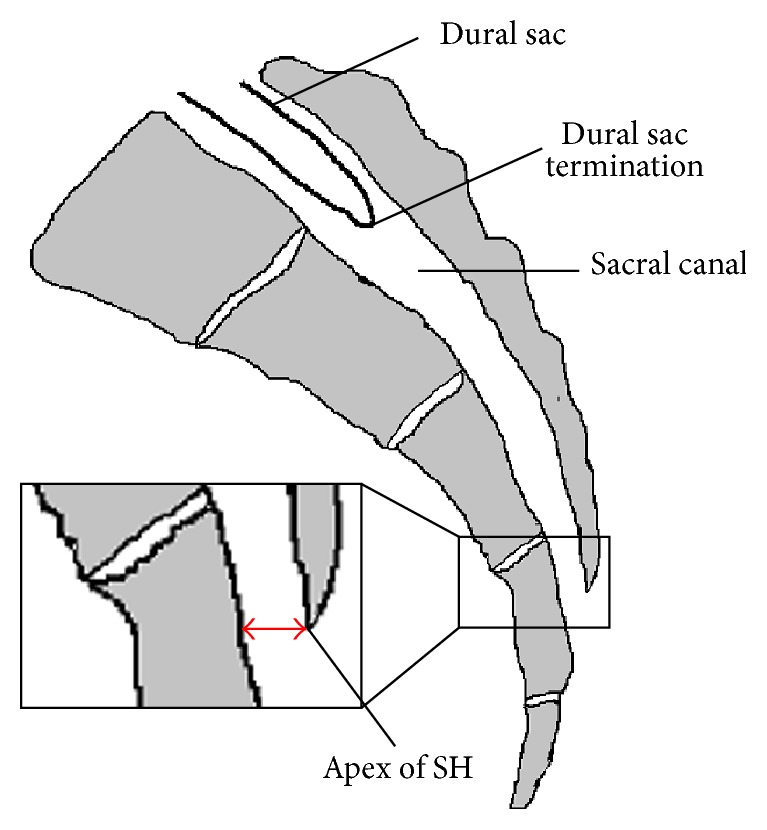
Sagittal view of sacrum. SH: sacral hiatus; red double-ended arrow: anterior-posterior diameter of sacral hiatus at its apex.

The anatomic features and variations relevant to caudal epidural block were the focuses of several recent reports. A thorough knowledge of the relevant anatomy (Figures 1 and 2) may improve the success rate of caudal epidural needle placement while minimize the risks of complications.

### 2.1. Sacral Cornua

The sacral cornua are vestigial remnants of the inferior articular processes of the 5th sacral vertebra and presented as two bony prominences at the caudal end of sacrum. Palpating the bilateral sacral cornua is essential to locate the sacral hiatus in the conventional landmark-based technique. However, the sacral cornua are not always palpable. Defining a height of at least 3 mm as palpable, Sekiguchi and colleagues reported that sacral cornua were bilaterally palpable in only 19%, unilaterally palpable in 25%, and bilaterally impalpable in 54% of isolated adult sacral bone [6]. Using the same definition, Aggarwal and colleagues reported that the sacral cornua were bilaterally palpable in 55%, unilaterally palpable in 24%, and bilaterally impalpable in 21% of adult sacral bone [7]. In another report, sacral cornua were not palpable bilaterally in 14.3% and palpable unilaterally in 24.5% of cadavers [8]. In a clinical report, sacral cornua were only palpable in 59% of individuals [4]. This high percentage of impalpable sacral cornua may be partially accountable for the high failure rate of the blind technique.

### 2.2. Sacral Hiatus

The sacral hiatus, resulting from failure of fusion of lamina and spinous process of lower sacral vertebrae, is the caudal termination of the sacral canal. The sacral hiatus is bordered laterally by two sacral cornua and could be palpable as a dimple in between. Posteriorly, the sacral hiatus is covered by the skin, subcutaneous fat, and sacrococcygeal ligament (SCL). During caudal epidural block, inserting a needle into the sacral hiatus is essential to access the sacral canal. However, certain anatomic features and variations of sacral hiatus may make it difficult or impossible to insert a needle into the caudal epidural space or predispose this procedure to complications such as dual puncture.

The mean anterior-posterior (AP) diameter of sacral hiatus at its apex ranges from 4.6 ± 2 mm to 6.1 ± 2.1 mm [6, 7, 9–14] and decreased with age [14]. In clinical settings, an AP diameter of sacral hiatus at the apex of less than 3.7 mm was associated with difficulty in inserting a needle into the caudal epidural space by blind technique [13]. When ultrasound is used to guide needle insertion, Chen and colleagues reported that difficulty was encountered in patients with the AP diameter of sacral hiatus at apex of less than 1.6 mm [11]. Similar result has been reported in another study using ultrasound guidance. In that study, the average AP diameter of sacral hiatus at apex in patients with failed caudal epidural needle insertion was 1.61 ± 0.1 mm, significantly shorter than that (4.7 ± 1.7 mm, *P* < 0.001) in patients with successful needle insertion [12]. The incidences of short AP diameter of sacral hiatus at its apex have been reported with different definitions. In studies using dry sacral bone, the sacral AP diameter was less than 3 mm in 8.77% [7] and less than 2 mm in 1%–6.25% of cases [6, 10]. In the extreme, the sacral hiatus is completely closed, precluding inserting a needle into the caudal epidural space via the sacral hiatus. The incidence of closed sacral hiatus was 2-3% from reports studying dry human sacral bone [6, 10].

### 2.3. Location of the Apex of the Sacral Hiatus

The apex of sacral hiatus is most commonly located at the S4 level (65–68%), followed by the S3 and S5 level (around 15% at each level) and the S1 to S2 level in 3–5% of cases [6, 8]. Complete agenesis of posterior wall of sacral canal (failure of fusion of sacral laminae) was noted in 1% of cases [6]. The higher the apex of sacral hiatus is located, the shorter the distance between it and the dural sac termination could be. Accidental dural puncture might occur if the needle is inserted near the apex of the sacral hiatus that is located at a high level of sacrum. On the other hand, the lower the apex of sacral hiatus is located, the shorter the length of the SCL could be. A length of the SCL of less than 17.6 mm was associated with difficult caudal epidural block by blind technique [13].

### 2.4. Dural Sac

The dural sac usually terminates between S1 and S2 vertebra, with the majority at S2 [8, 9, 15, 16]. In 1 to 5% of patients, the dural sac terminates at S3 or below [15, 16]. In addition, 1 to 5% of patients with low back pain or sciatica have a sacral Tarlov cyst [15–17], a perineural cyst that communicates with the dural sac and is filled with cerebrospinal fluid (CSF). More than 40% of the sacral Tarlov cysts are located at or below the S3 level [15, 16]. The lower the dural sac termination or the Tarlov cyst is located, the more likely dural puncture or intrathecal injection might occur during caudal epidural block.

### 2.5. Distance between the Dural Sac Termination and the Apex of the Sacral Hiatus

The distance between the dural sac termination and the apex of the sacral hiatus was the interest of several studies, because the risk of dural puncture is perceived to increase as this distance decreases. The average distance varies markedly from studies conducted in different ethnics. In an Indian cadaver study, the average distance was 32 ± 12 mm, ranging from 5.8 to 60.0 mm [8]. Using magnetic resonance imaging (MRI) for measurement, this distance was 60.3 ± 13.1 mm, ranging from 34 to 80 mm in a British study [9], and 44.6 ± 11.8 mm, ranging from 10 to 80 mm in a Turkish study [16]. As shown by these reports, the distance between the dural sac termination and the apex of the sacral hiatus could be as short as less than 6 mm in some individuals.

## 3. Techniques of Caudal Epidural Block

### 3.1. Blind Caudal Epidural Block

The patient can be placed in prone or lateral decubitus position for blind caudal epidural block. A line is draw to connect the bilateral posterior superior iliac crests and used as one side of an equilateral triangle; then the location of the sacral hiatus should be approximated. By palpating the sacral cornua as 2 bony prominences, the sacral hiatus could be identified as a dimple in between. A needle is inserted at 45 degrees to the sacrum and redirected if the posterior surface of sacral bone is contacted. A subjective feeling of “give” or loss of resistance suggests piercing the SCL [18] but is associated with a miss rate up to 26% even in experienced hands [5]. The “whoosh test,” performed by auscultation at the thoracolumbar region with a stethoscope while injecting 2 mL of air [19], has a sensitivity of 80% and a specificity of 60% in adults [20]. Palpating for subcutaneous bulging on rapid injection of 5 mL air or saline had a positive predictive value of 83% and a negative predictive value of 44% [4]. The inaccuracy of using blind technique for caudal epidural injection in adults, even confirmed by various tests, is clearly evident.

### 3.2. Fluoroscopy-Guided Caudal Epidural Block

**Figure 3:**
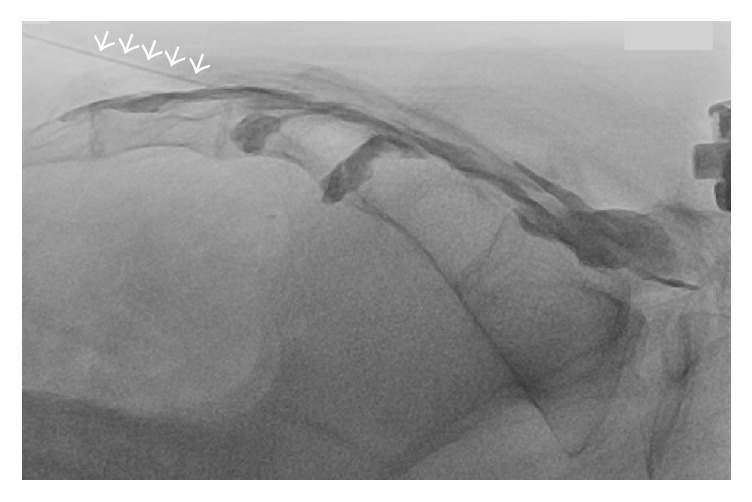
Fluoroscopy-guided caudal epidural block. Proper needle tip placement was verified by observing spread of contrast medium within the epidural space without intravascular uptake. Arrows: needle.

Because of the inaccuracy of blind technique, some authors have recommended that caudal epidural injection is performed under fluoroscopic guidance [3, 5]. The patient is usually placed in prone position for fluoroscopy-guided caudal epidural block. In lateral view of fluoroscopy, the sacral hiatus could be identified as an abrupt drop off at the end of S4 lamina [21]. The block needle trajectory can be visualized and navigated accordingly into the sacral canal. By injecting contrast medium under fluoroscopy, the placement of needle tip within the sacral epidural space can be verified (Figure 3), and intravascular or intrathecal needle tip placement can be detected. During caudal epidural injection, intravascular injection was reported in 3–14% of cases by conventional fluoroscopy even after negative aspiration [3, 22, 23]. Fluoroscopy guidance has markedly improved the successful rate of caudal epidural block [3–5, 23] and is now considered as the gold standard in performing caudal block. However, routine use of fluoroscopy for caudal epidural block is limited by radiation exposure, cost, and special space requirement.

### 3.3. Ultrasound-Guided Caudal Epidural Block

**Figure 4:**
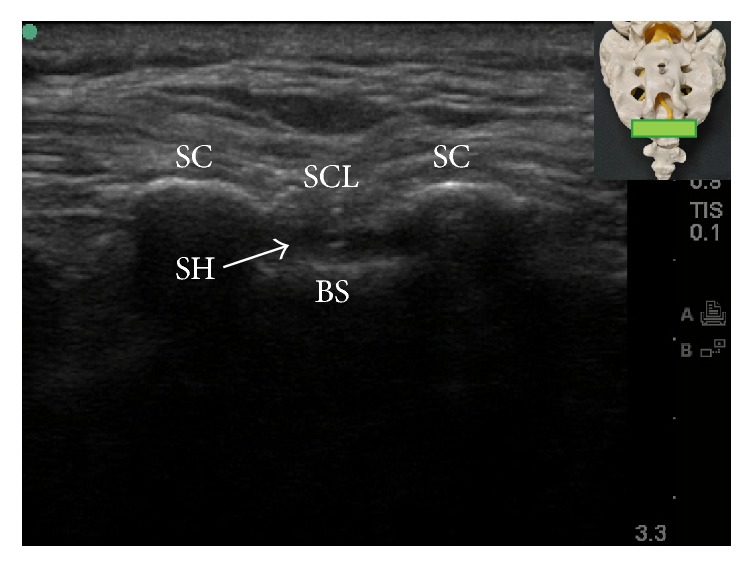
Transverse ultrasound view of the sacral hiatus. The inset shows the position of the ultrasound transducer. BS: base of sacrum; SC: sacral cornua; SCL: sacrococcygeal ligament; SH: sacral hiatus.

**Figure 5:**
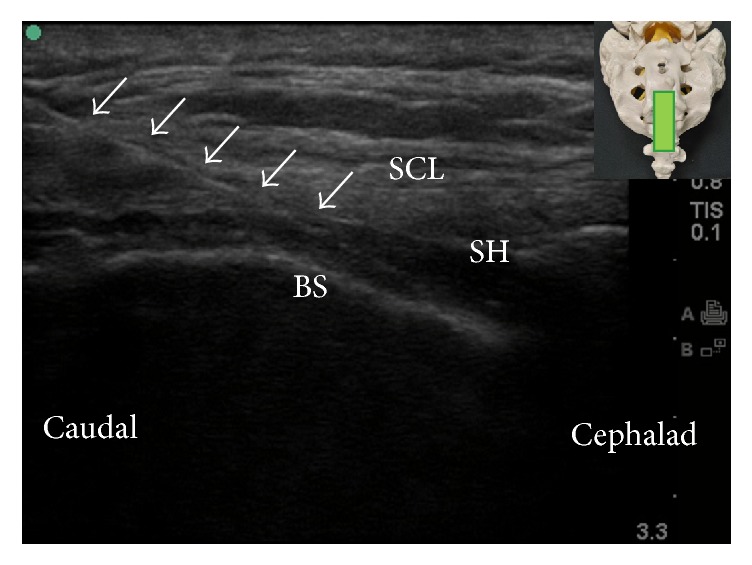
Longitudinal ultrasound view of sacral hiatus. The inset shows the position of the ultrasound transducer. BS: base of sacrum; SCL: sacrococcygeal ligament; SH: sacral hiatus; arrows: needle.

The ultrasound-guided caudal block was first described by Klocke and colleagues in 2003 [24] and has, since then, gained increasing popularity. Several studies from various ethnic populations have repeatedly reported very high successful rates (96.9–100%) of ultrasound-guided caudal injection [11, 12, 25–27]. The patient can be placed in prone or lateral decubitus position. Usually, a 7–13 MHz, liner transducer will suffice for most caudal epidural injection; however, a 2–5 MHz, curved transducer may be needed in obese patients. The ultrasound transducer was first placed transversely at the midline to obtain the transverse view of sacral hiatus (Figure 4). The two sacral cornua appear as two hyperechoic structures. Between the sacral cornua are two band-like hyperechoic structures; the superficial one is the SCL, and the deep one is the dorsal surface of sacral bone. The sacral hiatus was the hypoechoic region between the 2 band-like hyperechoic structures [25]. At this level, the ultrasound transducer is rotated 90 degrees to obtain the longitudinal view of sacral hiatus (Figure 5). Under longitudinal view, the block needle is inserted using the “in-plane” technique. The block needle can be visualized in real time, piercing the SCL, entering the sacral hiatus, but cannot be visualized beyond the apex of sacral hiatus. Therefore, without knowledge of dural sac termination from image study in advance, it is suggested that advancement of needle tip beyond the apex of sacral hiatus be limited to 5 mm to avoid dural puncture because the distance between the apex of sacral hiatus and dural sac termination can be as short as less than 6 mm [7].

**Figure 6:**
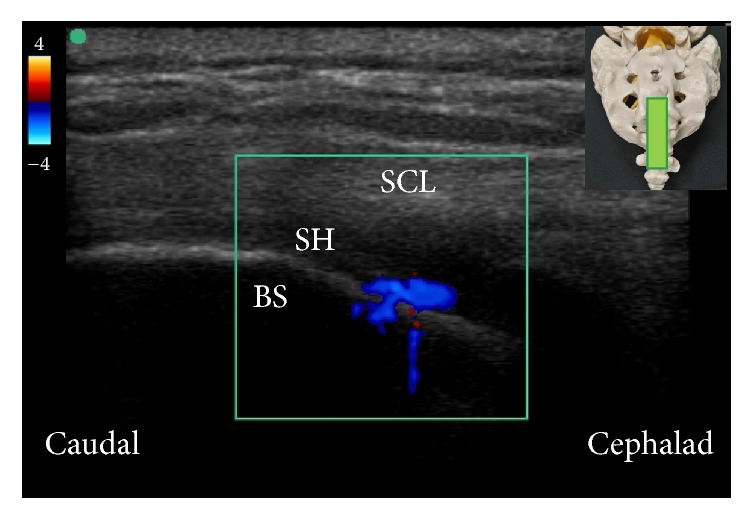
Color Doppler ultrasonography in longitudinal view of sacral hiatus. A predominantly one-color spectrum is observed in the sacral hiatus during caudal epidural injection. The inset shows the position of the ultrasound transducer. BS: base of sacrum; SCL: sacrococcygeal ligament; SH: sacral hiatus.

**Figure 7:**
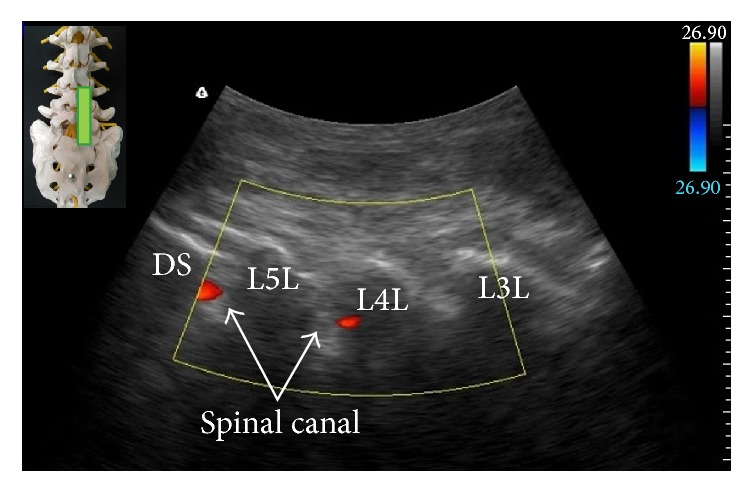
Color Doppler ultrasonography in paramedian sagittal oblique view of the sacral and lumbar spine. The observed color spectrum suggests the flow of injectate reaching L4-5 level. The inset shows the position of the ultrasound transducer. L3L: L3 lamina; L4L: L4 lamina; L5L: L5 lamina; DS: dorsal surface of sacrum.

Although ultrasonography cannot provide information regarding injectate spreading during caudal epidural injection as fluoroscopy, the presence of unidirectional flow, defined as one dominant color on color Doppler image, in the longitudinal view of sacral hiatus during injection (Figure 6) was reported to be predictive of successful caudal epidural injection [27, 28] and comparable treatment outcome as fluoroscopy-guided caudal epidural injection [28]. The ultrasonography could also provide information regarding the cephalad spreading of injectate during caudal epidural injection. Using a curved-array, low frequency (2–5 MHz) ultrasound transducer, the lumbar spinal canal could be visualized by the paramedian sagittal oblique view described by Chin and colleagues [29]. Observing color Doppler signal in the lumbar spinal canal during caudal epidural injection may indicate that the injectate has reached the lumbar epidural space (Figure 7), although this hypothesis needs to be confirmed in further studies.

While fluoroscopy with contrast medium injection is still considered the gold standard in preventing intravascular and intrathecal injection, ultrasonography could be, at least, as useful as fluoroscopy in preventing other complications during caudal epidural injection. For example, with the needle tip visualized real time going into the sacral hiatus by ultrasonography, advertently advancing the needle anteriorly into the rectum [30, 31] or a fetal skull in the birth canal [30] can be prevented. The practice of injecting air to verify needle tip position could be abandoned, because the injected air has been reported to cause portal vein air embolism [32] and motor weakness [33] after caudal epidural injection. In addition, ultrasound has some advantages over the fluoroscopy in guiding caudal epidural injection because it is easy to learn and radiation-free and can be virtually used in any clinical settings [25].

## 4. Conclusion

There are considerable anatomic variations relevant to caudal epidural block, which may contribute to failed block by landmark-based blind technique. The advent of fluoroscopy and ultrasound has markedly improved the successful rates of caudal epidural injection. Although fluoroscopy remains the gold standard in guiding caudal epidural injection, it is not always available and radiation exposure is a concern. In addition, routine use of fluoroscopy for caudal epidural injection seems impractical in the busy operating theater and office-based clinics. Given accumulating evidence has suggested that ultrasonography is excellent in guiding caudal epidural injection with similar treatment outcome as compared with fluoroscopy-guided caudal epidural injection, ultrasound should be the preferred alternative when fluoroscopy is not available.
